# Editorial: Causes for Increased Susceptibility to *Mycobacterium tuberculosis* – A Close View of the Immune System

**DOI:** 10.3389/fimmu.2015.00545

**Published:** 2015-10-22

**Authors:** Vishwanath Venketaraman

**Affiliations:** ^1^Department of Basic Medical Sciences, College of Osteopathic Medicine of the Pacific, Western University of Health Sciences, Pomona, CA, USA

**Keywords:** tuberculosis, host–pathogen interactions, host immune responses, pathogenesis, adaptive immunity

Worldwide, tuberculosis (TB) remains the most frequent and important infectious disease causing morbidity and death. One-third of the world’s population is infected with *Mycobacterium tuberculosis* (*M. tb*), the etiological agent of TB. The World Health Organization estimates that about 8–10 million new TB cases occur annually worldwide, and the incidence of TB is currently increasing. In this context, TB is in the top three (with malaria and HIV), being the leading cause of death from a single-infectious agent, and approximately three million deaths are attributable to TB annually. In particular, pulmonary TB, the most common form of TB, is a highly contagious and life-threatening disease. A major underlying factor responsible for increased susceptibility to *M. tb* is co-infection with HIV and the subsequent acquired immune deficiency syndrome. As of 2010, an estimated 34 million people are living with HIV infection worldwide, with an additional 2.7 million people newly infected each year. Of those 34 million living with HIV, 22.9 million live in sub-Saharan Africa, a region where *M. tb* is endemic. One of the hallmarks of AIDS brought on by HIV infection is increased susceptibility to opportunistic infections, including *M. tb*. Individuals with type II diabetes and cigarette smokers are also increasingly susceptible to *M. tb* infection. Successful control of *M. tb* infection requires effective innate and adaptive immune responses inside the granuloma leading to either killing or inhibition in the growth of *M. tb*. This Research Topic is a compilation of original research findings and review articles that are mainly centered on the pathogenesis of TB and host immune responses against *M. tb* infection. The articles in this Research Topic include

(a) Experimental findings describing how different subsets of primary human dendritic cells respond to *M. tb* infection (1).
(b) A comprehensive review on the variety of mechanisms by which mycobacteria subverts the host immune responses and intracellular effector mechanisms, leading to successful survival of the pathogen (2).
(c) Research article demonstrating that the mycobacterial gene, Rv1169c modifies the fatty acids in the cell wall of the pathogen, and induces necrosis in the host cells, thereby functioning as a virulence factor (3).
(d) A hypothetical and theory-based article describing the particle size distribution of cough aerosols as an important predictor of primary upper airway disease and cervical lymphadenitis in exposed hosts. The authors of this study hypothesize that large droplet aerosols (>5 μm) containing *M. tb* will be deposited in the upper airway and this in turn can induce host immune responses without establishing infection (4).
(e) Research findings that illustrate that IL-17 inhibits apoptosis of *M. bovis* BCG- or *M. tb*-infected macrophages thus hampering the ability of host cells to control bacterial growth (5).
(f) Review article describing the direct and indirect mechanisms by which natural killer cells control *M. tb* infection, and the effects of glutathione in improving the functions of natural killer cells to control *M. tb* infection (6).

## Conflict of Interest Statement

The author declares that the research was conducted in the absence of any commercial or financial relationships that could be construed as a potential conflict of interest.
